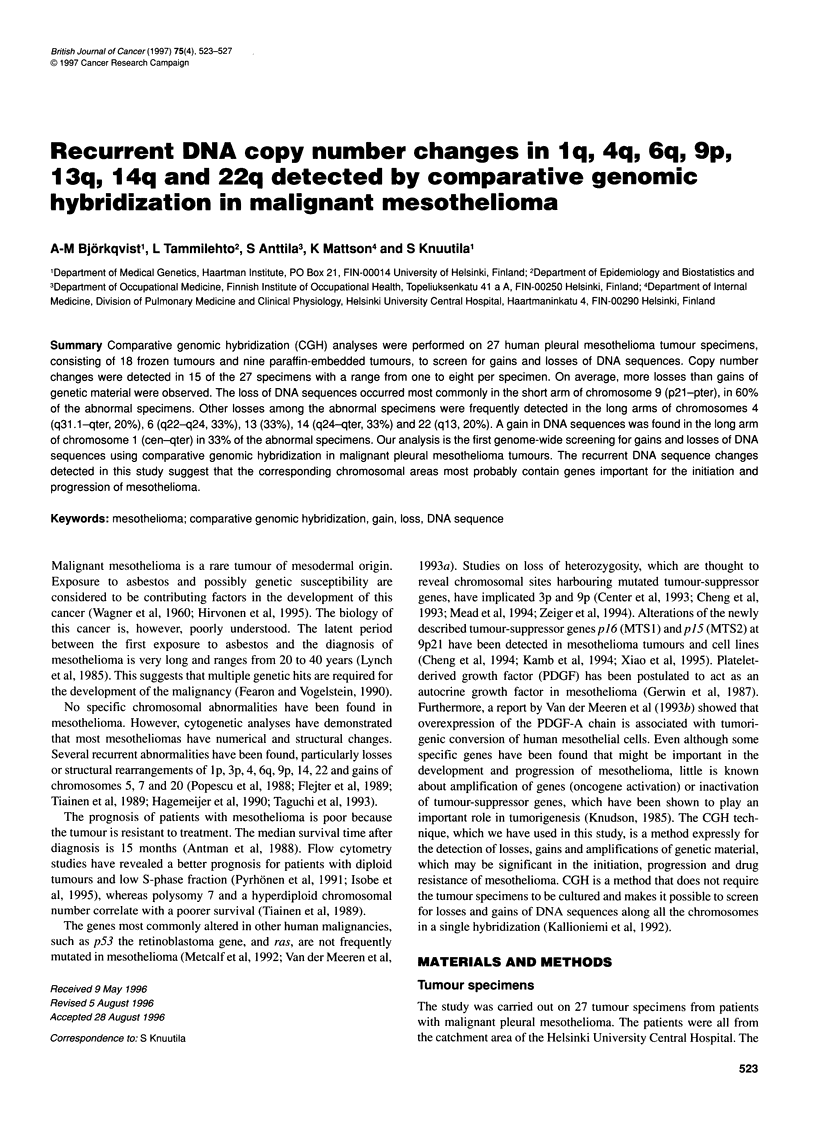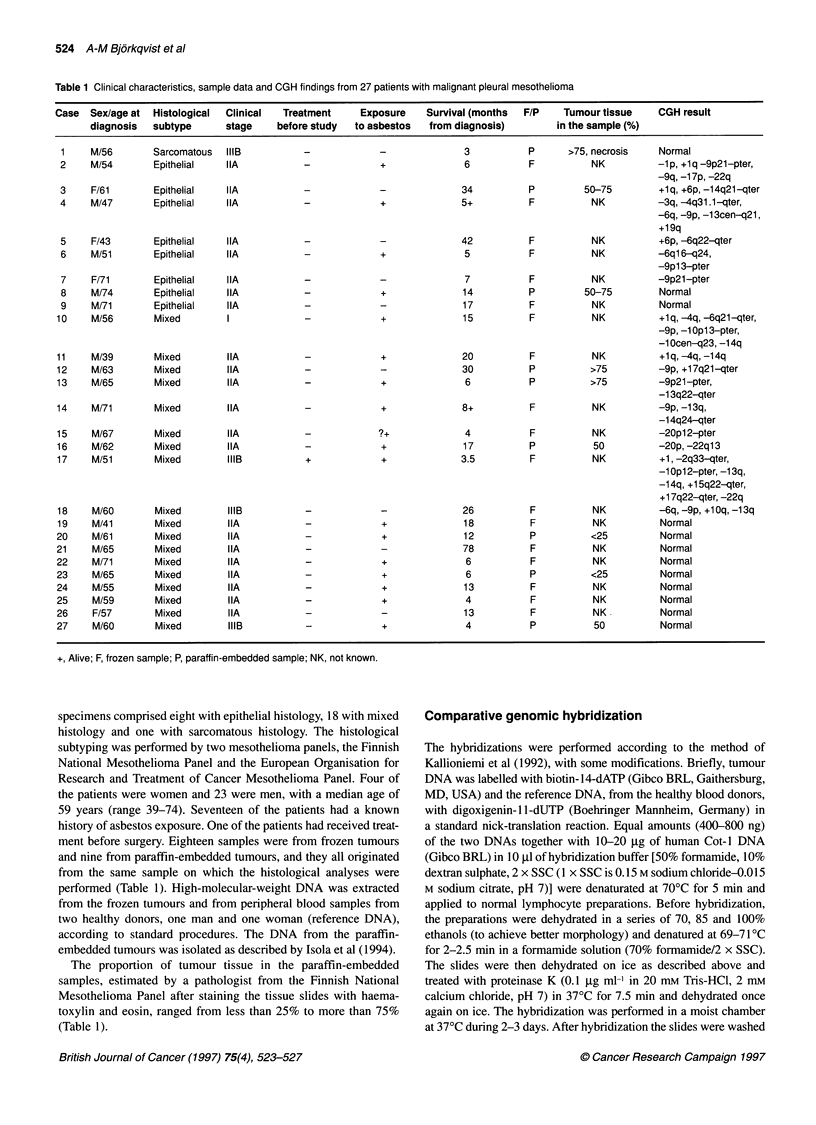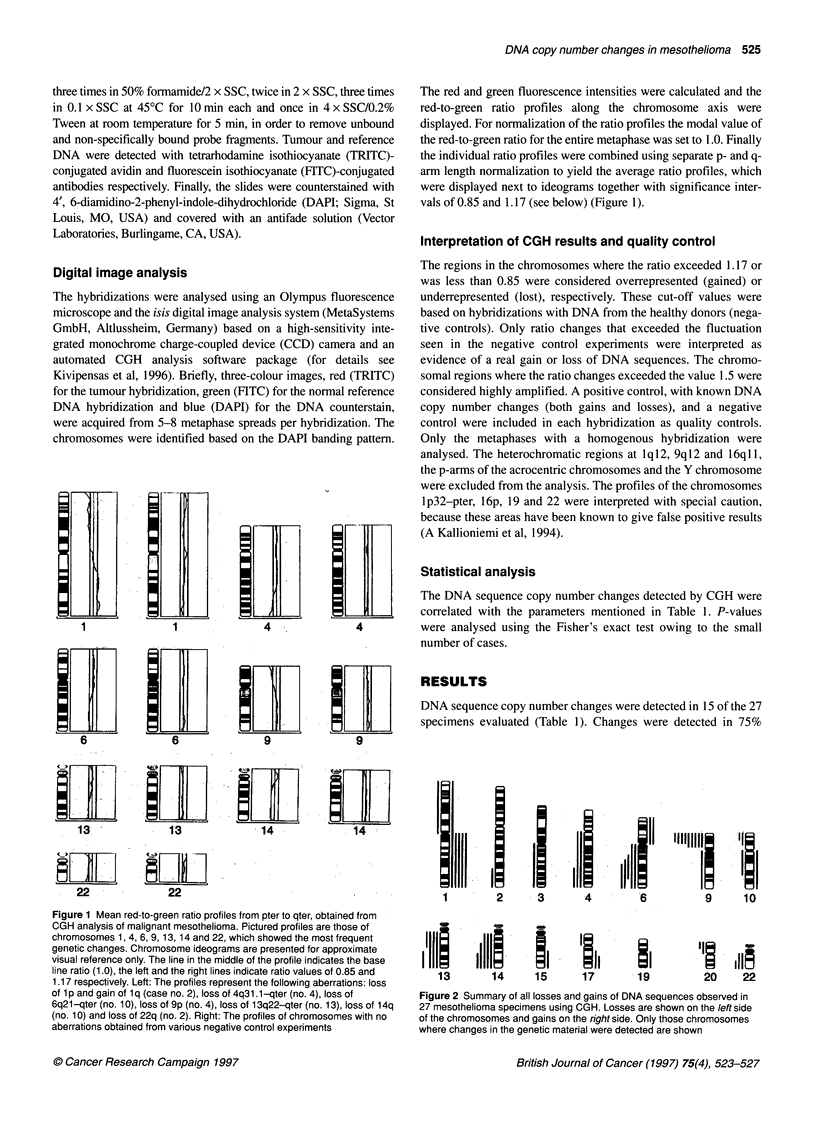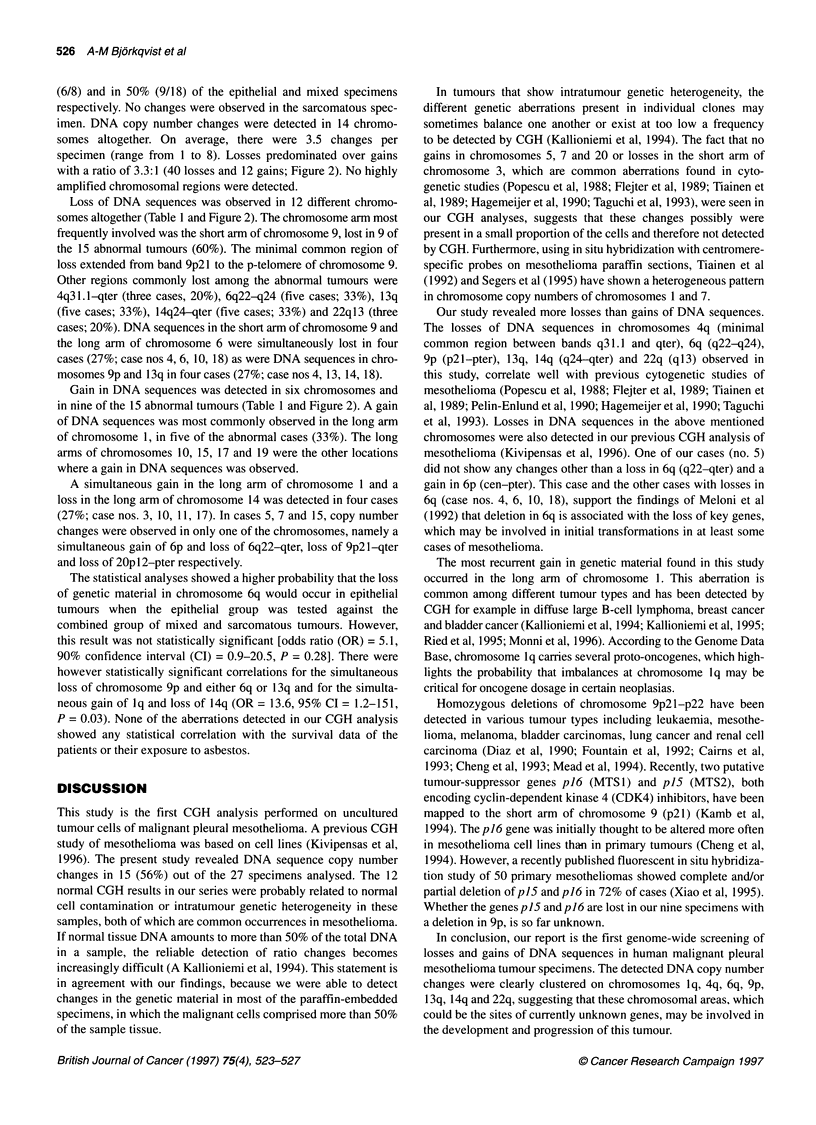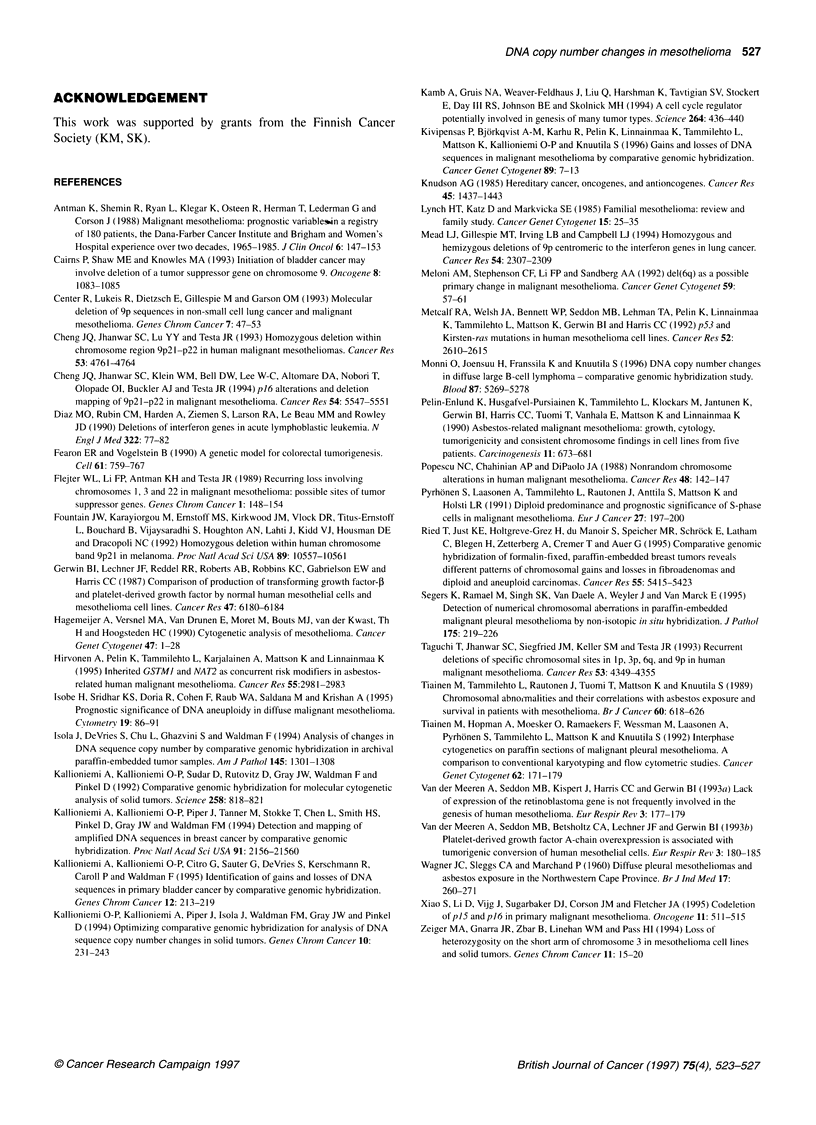# Recurrent DNA copy number changes in 1q, 4q, 6q, 9p, 13q, 14q and 22q detected by comparative genomic hybridization in malignant mesothelioma.

**DOI:** 10.1038/bjc.1997.91

**Published:** 1997

**Authors:** A. M. Björkqvist, L. Tammilehto, S. Anttila, K. Mattson, S. Knuutila

**Affiliations:** Department of Medical Genetics, Haartman Institute, University of Helsinki, Finland.

## Abstract

Comparative genomic hybridization (CGH) analyses were performed on 27 human pleural mesothelioma tumour specimens, consisting of 18 frozen tumours and nine paraffin-embedded tumours, to screen for gains and losses of DNA sequences. Copy number changes were detected in 15 of the 27 specimens with a range from one to eight per specimen. On average, more losses than gains of genetic material were observed. The loss of DNA sequences occurred most commonly in the short arm of chromosome 9 (p21-pter), in 60% of the abnormal specimens. Other losses among the abnormal specimens were frequently detected in the long arms of chromosomes 4 (q31.1-qter, 20%), 6 (q22-q24, 33%), 13 (33%),14 (q24-qter, 33%) and 22 (q13, 20%). A gain in DNA sequences was found in the long arm of chromosome 1 (cen-qter) in 33% of the abnormal specimens. Our analysis is the first genome-wide screening for gains and losses of DNA sequences using comparative genomic hybridization in malignant pleural mesothelioma tumours. The recurrent DNA sequence changes detected in this study suggest that the corresponding chromosomal areas most probably contain genes important for the initiation and progression of mesothelioma.


*British Journal of Cancer* (1997) **75**(4), 523–527
© 1997 Cancer Research Campaign

### Recurrent DNA copy number changes in 1q, 4q, 6q, 9p, 13q, 14q and 22q detected by comparative genomic hybridization in malignant mesothelioma

**A-M Björkqvist[1], L Tammilehto[2], S Anttila[3], K Mattson[4] and S Knuutila[1]**

[1]Department of Medical Genetics, Haartman Institute, PO Box 21, FIN-00014 University of Helsinki, Finland; [2]Department of Epidemiology and Biostatistics and [3]Department of Occupational Medicine, Finnish Institute of Occupational Health, Topeliuksenkatu 41 a A, FIN-00250 Helsinki, Finland; [4]Department of Internal Medicine, Division of Pulmonary Medicine and Clinical Physiology, Helsinki University Central Hospital, Haartmaninkatu 4, FIN-00290 Helsinki, Finland

**Summary** Comparative genomic hybridization (CGH) analyses were performed on 27 human pleural mesothelioma tumour specimens, consisting of 18 frozen tumours and nine paraffin-embedded tumours, to screen for gains and losses of DNA sequences. Copy number changes were detected in 15 of the 27 specimens with a range from one to eight per specimen. On average, more losses than gains of genetic material were observed. The loss of DNA sequences occurred most commonly in the short arm of chromosome 9 (p21–pter), in 60% of the abnormal specimens. Other losses among the abnormal specimens were frequently detected in the long arms of chromosomes 4 (q31.1–qter, 20%), 6 (q22–q24, 33%), 13 (33%), 14 (q24–qter, 33%) and 22 (q13, 20%). A gain in DNA sequences was found in the long arm of chromosome 1 (cen–qter) in 33% of the abnormal specimens. Our analysis is the first genome-wide screening for gains and losses of DNA sequences using comparative genomic hybridization in malignant pleural mesothelioma tumours. The recurrent DNA sequence changes detected in this study suggest that the corresponding chromosomal areas most probably contain genes important for the initiation and progression of mesothelioma.

**Keywords:** mesothelioma; comparative genomic hybridization, gain, loss, DNA sequence

Malignant mesothelioma is a rare tumour of mesodermal origin. Exposure to asbestos and possibly genetic susceptibility are considered to be contributing factors in the development of this cancer (Wagner et al, 1960; Hirvonen et al, 1995). The biology of this cancer is, however, poorly understood. The latent period between the first exposure to asbestos and the diagnosis of mesothelioma is very long and ranges from 20 to 40 years (Lynch et al, 1985). This suggests that multiple genetic hits are required for the development of the malignancy (Fearon and Vogelstein, 1990).

No specific chromosomal abnormalities have been found in mesothelioma. However, cytogenetic analyses have demonstrated that most mesotheliomas have numerical and structural changes. Several recurrent abnormalities have been found, particularly losses or structural rearrangements of 1p, 3p, 4, 6q, 9p, 14, 22 and gains of chromosomes 5, 7 and 20 (Popescu et al, 1988; Flejter et al, 1989; Tiainen et al, 1989; Hagemeijer et al, 1990; Taguchi et al, 1993).

The prognosis of patients with mesothelioma is poor because the tumour is resistant to treatment. The median survival time after diagnosis is 15 months (Antman et al, 1988). Flow cytometry studies have revealed a better prognosis for patients with diploid tumours and low S-phase fraction (Pyrhönen et al, 1991; Isobe et al, 1995), whereas polysomy 7 and a hyperdiploid chromosomal number correlate with a poorer survival (Tiainen et al, 1989).

The genes most commonly altered in other human malignancies, such as *p53* the retinoblastoma gene, and *ras*, are not frequently mutated in mesothelioma (Metcalf et al, 1992; Van der Meeren et al, 1993*a*). Studies on loss of heterozygosity, which are thought to reveal chromosomal sites harbouring mutated tumour-suppressor genes, have implicated 3p and 9p (Center et al, 1993; Cheng et al, 1993; Mead et al, 1994; Zeiger et al, 1994). Alterations of the newly described tumour-suppressor genes *p16* (MTS1) and *p15* (MTS2) at 9p21 have been detected in mesothelioma tumours and cell lines (Cheng et al, 1994; Kamb et al, 1994; Xiao et al, 1995). Platelet-derived growth factor (PDGF) has been postulated to act as an autocrine growth factor in mesothelioma (Gerwin et al, 1987). Furthermore, a report by Van der Meeren et al (1993*b*) showed that overexpression of the PDGF-A chain is associated with tumorigenic conversion of human mesothelial cells. Even although some specific genes have been found that might be important in the development and progression of mesothelioma, little is known about amplification of genes (oncogene activation) or inactivation of tumour-suppressor genes, which have been shown to play an important role in tumorigenesis (Knudson, 1985). The CGH technique, which we have used in this study, is a method expressly for the detection of losses, gains and amplifications of genetic material, which may be significant in the initiation, progression and drug resistance of mesothelioma. CGH is a method that does not require the tumour specimens to be cultured and makes it possible to screen for losses and gains of DNA sequences along all the chromosomes in a single hybridization (Kallioniemi et al, 1992).

*Received 9 May 1996*
*Revised 5 August 1996*
*Accepted 28 August 1996*

*Correspondence to:* S Knuutila

### MATERIALS AND METHODS

### Tumour specimens

The study was carried out on 27 tumour specimens from patients with malignant pleural mesothelioma. The patients were all from the catchment area of the Helsinki University Central Hospital. The

**Table 1** Clinical characteristics, sample data and CGH findings from 27 patients with malignant pleural mesothelioma

| Case | Sex/age at diagnosis | Histological subtype | Clinical stage | Treatment before study | Exposure to asbestos | Survival (months from diagnosis) | F/P | Tumour tissue in the sample (%) | CGH result |
|---|---|---|---|---|---|---|---|---|---|
| 1 | M/56 | Sarcomatous | IIIB | – | – | 3 | P | >75, necrosis | Normal |
| 2 | M/54 | Epithelial | IIA | – | + | 6 | F | NK | –1p, +1q –9p21–pter, –9q, –17p, –22q |
| 3 | F/61 | Epithelial | IIA | – | – | 34 | P | 50–75 | +1q, +6p, –14q21–qter |
| 4 | M/47 | Epithelial | IIA | – | + | 5+ | F | NK | –3q, –4q31.1–qter, –6q, –9p, –13cen–q21, +19q |
| 5 | F/43 | Epithelial | IIA | – | – | 42 | F | NK | +6p, –6q22–qter |
| 6 | M/51 | Epithelial | IIA | – | + | 5 | F | NK | –6q16–q24, –9p13–pter |
| 7 | F/71 | Epithelial | IIA | – | – | 7 | F | NK | –9p21–pter |
| 8 | M/74 | Epithelial | IIA | – | + | 14 | P | 50–75 | Normal |
| 9 | M/71 | Epithelial | IIA | – | – | 17 | F | NK | Normal |
| 10 | M/56 | Mixed | I | – | + | 15 | F | NK | +1q, –4q, –6q21–qter, –9p, –10p13–pter, –10cen–q23, –14q |
| 11 | M/39 | Mixed | IIA | – | + | 20 | F | NK | +1q, –4q, –14q |
| 12 | M/63 | Mixed | IIA | – | – | 30 | P | >75 | –9p, +17q21–qter |
| 13 | M/65 | Mixed | IIA | – | + | 6 | P | >75 | –9p21–pter, –13q22–qter |
| 14 | M/71 | Mixed | IIA | – | + | 8+ | F | NK | –9p, –13q, –14q24–qter |
| 15 | M/67 | Mixed | IIA | – | ?+ | 4 | F | NK | –20p12–pter |
| 16 | M/62 | Mixed | IIA | – | + | 17 | P | 50 | –20p, –22q13 |
| 17 | M/51 | Mixed | IIIB | + | + | 3.5 | F | NK | +1, –2q33–qter, –10p12–pter, –13q, –14q, +15q22–qter, +17q22–qter, –22q |
| 18 | M/60 | Mixed | IIIB | – | – | 26 | F | NK | –6q, –9p, +10q, –13q |
| 19 | M/41 | Mixed | IIA | – | + | 18 | F | NK | Normal |
| 20 | M/61 | Mixed | IIA | – | + | 12 | P | <25 | Normal |
| 21 | M/65 | Mixed | IIA | – | – | 78 | F | NK | Normal |
| 22 | M/71 | Mixed | IIA | – | + | 6 | F | NK | Normal |
| 23 | M/65 | Mixed | IIA | – | + | 6 | P | <25 | Normal |
| 24 | M/55 | Mixed | IIA | – | + | 13 | F | NK | Normal |
| 25 | M/59 | Mixed | IIA | – | + | 4 | F | NK | Normal |
| 26 | F/57 | Mixed | IIA | – | – | 13 | F | NK | Normal |
| 27 | M/60 | Mixed | IIIB | – | + | 4 | P | 50 | Normal |

+, Alive; F, frozen sample; P, paraffin-embedded sample; NK, not known.

specimens comprised eight with epithelial histology, 18 with mixed histology and one with sarcomatous histology. The histological subtyping was performed by two mesothelioma panels, the Finnish National Mesothelioma Panel and the European Organisation for Research and Treatment of Cancer Mesothelioma Panel. Four of the patients were women and 23 were men, with a median age of 59 years (range 39–74). Seventeen of the patients had a known history of asbestos exposure. One of the patients had received treatment before surgery. Eighteen samples were from frozen tumours and nine from paraffin-embedded tumours, and they all originated from the same sample on which the histological analyses were performed (Table 1). High-molecular-weight DNA was extracted from the frozen tumours and from peripheral blood samples from two healthy donors, one man and one woman (reference DNA), according to standard procedures. The DNA from the paraffin-embedded tumours was isolated as described by Isola et al (1994).

The proportion of tumour tissue in the paraffin-embedded samples, estimated by a pathologist from the Finnish National Mesothelioma Panel after staining the tissue slides with haematoxylin and eosin, ranged from less than 25% to more than 75% (Table 1).

### Comparative genomic hybridization

The hybridizations were performed according to the method of Kallioniemi et al (1992), with some modifications. Briefly, tumour DNA was labelled with biotin-14-dATP (Gibco BRL, Gaithersburg, MD, USA) and the reference DNA, from the healthy blood donors, with digoxigenin-11-dUTP (Boehringer Mannheim, Germany) in a standard nick-translation reaction. Equal amounts (400–800 ng) of the two DNAs together with 10–20 μg of human Cot-1 DNA (Gibco BRL) in 10 μl of hybridization buffer [50% formamide, 10% dextran sulphate, 2 × SSC (1 × SSC is 0.15 M sodium chloride–0.015 M sodium citrate, pH 7)] were denaturated at 70°C for 5 min and applied to normal lymphocyte preparations. Before hybridization, the preparations were dehydrated in a series of 70, 85 and 100% ethanols (to achieve better morphology) and denatured at 69–71°C for 2–2.5 min in a formamide solution (70% formamide/2 × SSC). The slides were then dehydrated on ice as described above and treated with proteinase K (0.1 μg $ml^{-1}$ in 20 mM Tris-HCl, 2 mM calcium chloride, pH 7) in 37°C for 7.5 min and dehydrated once again on ice. The hybridization was performed in a moist chamber at 37°C during 2–3 days. After hybridization the slides were washed

 © Cancer Research Campaign 1997

three times in 50% formamide/2 × SSC, twice in 2 × SSC, three times in 0.1 × SSC at 45°C for 10 min each and once in 4 × SSC/0.2% Tween at room temperature for 5 min, in order to remove unbound and non-specifically bound probe fragments. Tumour and reference DNA were detected with tetrarhodamine isothiocyanate (TRITC)-conjugated avidin and fluorescein isothiocyanate (FITC)-conjugated antibodies respectively. Finally, the slides were counterstained with 4′, 6-diamidino-2-phenyl-indole-dihydrochloride (DAPI; Sigma, St Louis, MO, USA) and covered with an antifade solution (Vector Laboratories, Burlingame, CA, USA).

### Digital image analysis

The hybridizations were analysed using an Olympus fluorescence microscope and the *isis* digital image analysis system (MetaSystems GmbH, Altlussheim, Germany) based on a high-sensitivity integrated monochrome charge-coupled device (CCD) camera and an automated CGH analysis software package (for details see Kivipensas et al, 1996). Briefly, three-colour images, red (TRITC) for the tumour hybridization, green (FITC) for the normal reference DNA hybridization and blue (DAPI) for the DNA counterstain, were acquired from 5–8 metaphase spreads per hybridization. The chromosomes were identified based on the DAPI banding pattern.

**Figure 1** Mean red-to-green ratio profiles from pter to qter, obtained from CGH analysis of malignant mesothelioma. Pictured profiles are those of chromosomes 1, 4, 6, 9, 13, 14 and 22, which showed the most frequent genetic changes. Chromosome ideograms are presented for approximate visual reference only. The line in the middle of the profile indicates the base line ratio (1.0), the left and the right lines indicate ratio values of 0.85 and 1.17 respectively. Left: The profiles represent the following aberrations: loss of 1p and gain of 1q (case no. 2), loss of 4q31.1–qter (no. 4), loss of 6q21–qter (no. 10), loss of 9p (no. 4), loss of 13q22–qter (no. 13), loss of 14q (no. 10) and loss of 22q (no. 2). Right: The profiles of chromosomes with no aberrations obtained from various negative control experiments

The red and green fluorescence intensities were calculated and the red-to-green ratio profiles along the chromosome axis were displayed. For normalization of the ratio profiles the modal value of the red-to-green ratio for the entire metaphase was set to 1.0. Finally the individual ratio profiles were combined using separate p- and q-arm length normalization to yield the average ratio profiles, which were displayed next to ideograms together with significance intervals of 0.85 and 1.17 (see below) (Figure 1).

### Interpretation of CGH results and quality control

The regions in the chromosomes where the ratio exceeded 1.17 or was less than 0.85 were considered overrepresented (gained) or underrepresented (lost), respectively. These cut-off values were based on hybridizations with DNA from the healthy donors (negative controls). Only ratio changes that exceeded the fluctuation seen in the negative control experiments were interpreted as evidence of a real gain or loss of DNA sequences. The chromosomal regions where the ratio changes exceeded the value 1.5 were considered highly amplified. A positive control, with known DNA copy number changes (both gains and losses), and a negative control were included in each hybridization as quality controls. Only the metaphases with a homogenous hybridization were analysed. The heterochromatic regions at 1q12, 9q12 and 16q11, the p-arms of the acrocentric chromosomes and the Y chromosome were excluded from the analysis. The profiles of the chromosomes 1p32–pter, 16p, 19 and 22 were interpreted with special caution, because these areas have been known to give false positive results (A Kallioniemi et al, 1994).

### Statistical analysis

The DNA sequence copy number changes detected by CGH were correlated with the parameters mentioned in Table 1. *P*-values were analysed using the Fisher's exact test owing to the small number of cases.

### RESULTS

DNA sequence copy number changes were detected in 15 of the 27 specimens evaluated (Table 1). Changes were detected in 75%

**Figure 2** Summary of all losses and gains of DNA sequences observed in 27 mesothelioma specimens using CGH. Losses are shown on the *left* side of the chromosomes and gains on the *right* side. Only those chromosomes where changes in the genetic material were detected are shown

© Cancer Research Campaign 1997

(6/8) and in 50% (9/18) of the epithelial and mixed specimens respectively. No changes were observed in the sarcomatous specimen. DNA copy number changes were detected in 14 chromosomes altogether. On average, there were 3.5 changes per specimen (range from 1 to 8). Losses predominated over gains with a ratio of 3.3:1 (40 losses and 12 gains; Figure 2). No highly amplified chromosomal regions were detected.

Loss of DNA sequences was observed in 12 different chromosomes altogether (Table 1 and Figure 2). The chromosome arm most frequently involved was the short arm of chromosome 9, lost in 9 of the 15 abnormal tumours (60%). The minimal common region of loss extended from band 9p21 to the p-telomere of chromosome 9. Other regions commonly lost among the abnormal tumours were 4q31.1–qter (three cases, 20%), 6q22–q24 (five cases; 33%), 13q (five cases; 33%), 14q24–qter (five cases; 33%) and 22q13 (three cases; 20%). DNA sequences in the short arm of chromosome 9 and the long arm of chromosome 6 were simultaneously lost in four cases (27%; case nos 4, 6, 10, 18) as were DNA sequences in chromosomes 9p and 13q in four cases (27%; case nos 4, 13, 14, 18).

Gain in DNA sequences was detected in six chromosomes and in nine of the 15 abnormal tumours (Table 1 and Figure 2). A gain of DNA sequences was most commonly observed in the long arm of chromosome 1, in five of the abnormal cases (33%). The long arms of chromosomes 10, 15, 17 and 19 were the other locations where a gain in DNA sequences was observed.

A simultaneous gain in the long arm of chromosome 1 and a loss in the long arm of chromosome 14 was detected in four cases (27%; case nos. 3, 10, 11, 17). In cases 5, 7 and 15, copy number changes were observed in only one of the chromosomes, namely a simultaneous gain of 6p and loss of 6q22–qter, loss of 9p21–qter and loss of 20p12–pter respectively.

The statistical analyses showed a higher probability that the loss of genetic material in chromosome 6q would occur in epithelial tumours when the epithelial group was tested against the combined group of mixed and sarcomatous tumours. However, this result was not statistically significant [odds ratio (OR) = 5.1, 90% confidence interval (CI) = 0.9–20.5, $P$ = 0.28]. There were however statistically significant correlations for the simultaneous loss of chromosome 9p and either 6q or 13q and for the simultaneous gain of 1q and loss of 14q (OR = 13.6, 95% CI = 1.2–151, $P$ = 0.03). None of the aberrations detected in our CGH analysis showed any statistical correlation with the survival data of the patients or their exposure to asbestos.

### DISCUSSION

This study is the first CGH analysis performed on uncultured tumour cells of malignant pleural mesothelioma. A previous CGH study of mesothelioma was based on cell lines (Kivipensas et al, 1996). The present study revealed DNA sequence copy number changes in 15 (56%) out of the 27 specimens analysed. The 12 normal CGH results in our series were probably related to normal cell contamination or intratumour genetic heterogeneity in these samples, both of which are common occurrences in mesothelioma. If normal tissue DNA amounts to more than 50% of the total DNA in a sample, the reliable detection of ratio changes becomes increasingly difficult (A Kallioniemi et al, 1994). This statement is in agreement with our findings, because we were able to detect changes in the genetic material in most of the paraffin-embedded specimens, in which the malignant cells comprised more than 50% of the sample tissue.

In tumours that show intratumour genetic heterogeneity, the different genetic aberrations present in individual clones may sometimes balance one another or exist at too low a frequency to be detected by CGH (Kallioniemi et al, 1994). The fact that no gains in chromosomes 5, 7 and 20 or losses in the short arm of chromosome 3, which are common aberrations found in cytogenetic studies (Popescu et al, 1988; Flejter et al, 1989; Tiainen et al, 1989; Hagemeijer et al, 1990; Taguchi et al, 1993), were seen in our CGH analyses, suggests that these changes possibly were present in a small proportion of the cells and therefore not detected by CGH. Furthermore, using in situ hybridization with centromere-specific probes on mesothelioma paraffin sections, Tiainen et al (1992) and Segers et al (1995) have shown a heterogeneous pattern in chromosome copy numbers of chromosomes 1 and 7.

Our study revealed more losses than gains of DNA sequences. The losses of DNA sequences in chromosomes 4q (minimal common region between bands q31.1 and qter), 6q (q22–q24), 9p (p21–pter), 13q, 14q (q24–qter) and 22q (q13) observed in this study, correlate well with previous cytogenetic studies of mesothelioma (Popescu et al, 1988; Flejter et al, 1989; Tiainen et al, 1989; Pelin-Enlund et al, 1990; Hagemeijer et al, 1990; Taguchi et al, 1993). Losses in DNA sequences in the above mentioned chromosomes were also detected in our previous CGH analysis of mesothelioma (Kivipensas et al, 1996). One of our cases (no. 5) did not show any changes other than a loss in 6q (q22–qter) and a gain in 6p (cen–pter). This case and the other cases with losses in 6q (case nos. 4, 6, 10, 18), support the findings of Meloni et al (1992) that deletion in 6q is associated with the loss of key genes, which may be involved in initial transformations in at least some cases of mesothelioma.

The most recurrent gain in genetic material found in this study occurred in the long arm of chromosome 1. This aberration is common among different tumour types and has been detected by CGH for example in diffuse large B-cell lymphoma, breast cancer and bladder cancer (Kallioniemi et al, 1994; Kallioniemi et al, 1995; Ried et al, 1995; Monni et al, 1996). According to the Genome Data Base, chromosome 1q carries several proto-oncogenes, which highlights the probability that imbalances at chromosome 1q may be critical for oncogene dosage in certain neoplasias.

Homozygous deletions of chromosome 9p21–p22 have been detected in various tumour types including leukaemia, mesothelioma, melanoma, bladder carcinomas, lung cancer and renal cell carcinoma (Diaz et al, 1990; Fountain et al, 1992; Cairns et al, 1993; Cheng et al, 1993; Mead et al, 1994). Recently, two putative tumour-suppressor genes *p16* (MTS1) and *p15* (MTS2), both encoding cyclin-dependent kinase 4 (CDK4) inhibitors, have been mapped to the short arm of chromosome 9 (p21) (Kamb et al, 1994). The *p16* gene was initially thought to be altered more often in mesothelioma cell lines than in primary tumours (Cheng et al, 1994). However, a recently published fluorescent in situ hybridization study of 50 primary mesotheliomas showed complete and/or partial deletion of *p15* and *p16* in 72% of cases (Xiao et al, 1995). Whether the genes *p15* and *p16* are lost in our nine specimens with a deletion in 9p, is so far unknown.

In conclusion, our report is the first genome-wide screening of losses and gains of DNA sequences in human malignant pleural mesothelioma tumour specimens. The detected DNA copy number changes were clearly clustered on chromosomes 1q, 4q, 6q, 9p, 13q, 14q and 22q, suggesting that these chromosomal areas, which could be the sites of currently unknown genes, may be involved in the development and progression of this tumour.

© Cancer Research Campaign 1997

### ACKNOWLEDGEMENT

This work was supported by grants from the Finnish Cancer Society (KM, SK).

### REFERENCES

Antman K, Shemin R, Ryan L, Klegar K, Osteen R, Herman T, Lederman G and Corson J (1988) Malignant mesothelioma: prognostic variables in a registry of 180 patients, the Dana-Farber Cancer Institute and Brigham and Women's Hospital experience over two decades, 1965–1985. *J Clin Oncol* **6**: 147–153

Cairns P, Shaw ME and Knowles MA (1993) Initiation of bladder cancer may involve deletion of a tumor suppressor gene on chromosome 9. *Oncogene* **8**: 1083–1085

Center R, Lukeis R, Dietzsch E, Gillespie M and Garson OM (1993) Molecular deletion of 9p sequences in non-small cell lung cancer and malignant mesothelioma. *Genes Chrom Cancer* **7**: 47–53

Cheng JQ, Jhanwar SC, Lu YY and Testa JR (1993) Homozygous deletion within chromosome region 9p21–p22 in human malignant mesotheliomas. *Cancer Res* **53**: 4761–4764

Cheng JQ, Jhanwar SC, Klein WM, Bell DW, Lee W-C, Altomare DA, Nobori T, Olopade OI, Buckler AJ and Testa JR (1994) *p16* alterations and deletion mapping of 9p21–p22 in malignant mesothelioma. *Cancer Res* **54**: 5547–5551

Diaz MO, Rubin CM, Harden A, Ziemen S, Larson RA, Le Beau MM and Rowley JD (1990) Deletions of interferon genes in acute lymphoblastic leukemia. *N Engl J Med* **322**: 77–82

Fearon ER and Vogelstein B (1990) A genetic model for colorectal tumorigenesis. *Cell* **61**: 759–767

Flejter WL, Li FP, Antman KH and Testa JR (1989) Recurring loss involving chromosomes 1, 3 and 22 in malignant mesothelioma: possible sites of tumor suppressor genes. *Genes Chrom Cancer* **1**: 148–154

Fountain JW, Karayiorgou M, Ernstoff MS, Kirkwood JM, Vlock DR, Titus-Ernstoff L, Bouchard B, Vijaysaradhi S, Houghton AN, Lahti J, Kidd VJ, Housman DE and Dracopoli NC (1992) Homozygous deletion within human chromosome band 9p21 in melanoma. *Proc Natl Acad Sci USA* **89**: 10557–10561

Gerwin BI, Lechner JF, Reddel RR, Roberts AB, Robbins KC, Gabrielson EW and Harris CC (1987) Comparison of production of transforming growth factor-β and platelet-derived growth factor by normal human mesothelial cells and mesothelioma cell lines. *Cancer Res* **47**: 6180–6184

Hagemeijer A, Versnel MA, Van Drunen E, Moret M, Bouts MJ, van der Kwast, Th H and Hoogsteden HC (1990) Cytogenetic analysis of mesothelioma. *Cancer Genet Cytogenet* **47**: 1–28

Hirvonen A, Pelin K, Tammilehto L, Karjalainen A, Mattson K and Linnainmaa K (1995) Inherited *GSTM1* and *NAT2* as concurrent risk modifiers in asbestos-related human malignant mesothelioma. *Cancer Res* **55**:2981–2983

Isobe H, Sridhar KS, Doria R, Cohen F, Raub WA, Saldana M and Krishan A (1995) Prognostic significance of DNA aneuploidy in diffuse malignant mesothelioma. *Cytometry* **19**: 86–91

Isola J, DeVries S, Chu L, Ghazvini S and Waldman F (1994) Analysis of changes in DNA sequence copy number by comparative genomic hybridization in archival paraffin-embedded tumor samples. *Am J Pathol* **145**: 1301–1308

Kallioniemi A, Kallioniemi O-P, Sudar D, Rutovitz D, Gray JW, Waldman F and Pinkel D (1992) Comparative genomic hybridization for molecular cytogenetic analysis of solid tumors. *Science* **258**: 818–821

Kallioniemi A, Kallioniemi O-P, Piper J, Tanner M, Stokke T, Chen L, Smith HS, Pinkel D, Gray JW and Waldman FM (1994) Detection and mapping of amplified DNA sequences in breast cancer by comparative genomic hybridization. *Proc Natl Acad Sci USA* **91**: 2156–21560

Kallioniemi A, Kallioniemi O-P, Citro G, Sauter G, DeVries S, Kerschmann R, Caroll P and Waldman F (1995) Identification of gains and losses of DNA sequences in primary bladder cancer by comparative genomic hybridization. *Genes Chrom Cancer* **12**: 213–219

Kallioniemi O-P, Kallioniemi A, Piper J, Isola J, Waldman FM, Gray JW and Pinkel D (1994) Optimizing comparative genomic hybridization for analysis of DNA sequence copy number changes in solid tumors. *Genes Chrom Cancer* **10**: 231–243

Kamb A, Gruis NA, Weaver-Feldhaus J, Liu Q, Harshman K, Tavtigian SV, Stockert E, Day III RS, Johnson BE and Skolnick MH (1994) A cell cycle regulator potentially involved in genesis of many tumor types. *Science* **264**: 436–440

Kivipensas P, Björkqvist A-M, Karhu R, Pelin K, Linnainmaa K, Tammilehto L, Mattson K, Kallioniemi O-P and Knuutila S (1996) Gains and losses of DNA sequences in malignant mesothelioma by comparative genomic hybridization. *Cancer Genet Cytogenet* **89**: 7–13

Knudson AG (1985) Hereditary cancer, oncogenes, and antioncogenes. *Cancer Res* **45**: 1437–1443

Lynch HT, Katz D and Markvicka SE (1985) Familial mesothelioma: review and family study. *Cancer Genet Cytogenet* **15**: 25–35

Mead LJ, Gillespie MT, Irving LB and Campbell LJ (1994) Homozygous and hemizygous deletions of 9p centromeric to the interferon genes in lung cancer. *Cancer Res* **54**: 2307–2309

Meloni AM, Stephenson CF, Li FP and Sandberg AA (1992) del(6q) as a possible primary change in malignant mesothelioma. *Cancer Genet Cytogenet* **59**: 57–61

Metcalf RA, Welsh JA, Bennett WP, Seddon MB, Lehman TA, Pelin K, Linnainmaa K, Tammilehto L, Mattson K, Gerwin BI and Harris CC (1992) *p53* and Kirsten-*ras* mutations in human mesothelioma cell lines. *Cancer Res* **52**: 2610–2615

Monni O, Joensuu H, Franssila K and Knuutila S (1996) DNA copy number changes in diffuse large B-cell lymphoma – comparative genomic hybridization study. *Blood* **87**: 5269–5278

Pelin-Enlund K, Husgafvel-Pursiainen K, Tammilehto L, Klockars M, Jantunen K, Gerwin BI, Harris CC, Tuomi T, Vanhala E, Mattson K and Linnainmaa K (1990) Asbestos-related malignant mesothelioma: growth, cytology, tumorigenicity and consistent chromosome findings in cell lines from five patients. *Carcinogenesis* **11**: 673–681

Popescu NC, Chahinian AP and DiPaolo JA (1988) Nonrandom chromosome alterations in human malignant mesothelioma. *Cancer Res* **48**: 142–147

Pyrhönen S, Laasonen A, Tammilehto L, Rautonen J, Anttila S, Mattson K and Holsti LR (1991) Diploid predominance and prognostic significance of S-phase cells in malignant mesothelioma. *Eur J Cancer* **27**: 197–200

Ried T, Just KE, Holtgreve-Grez H, du Manoir S, Speicher MR, Schröck E, Latham C, Blegen H, Zetterberg A, Cremer T and Auer G (1995) Comparative genomic hybridization of formalin-fixed, paraffin-embedded breast tumors reveals different patterns of chromosomal gains and losses in fibroadenomas and diploid and aneuploid carcinomas. *Cancer Res* **55**: 5415–5423

Segers K, Ramael M, Singh SK, Van Daele A, Weyler J and Van Marck E (1995) Detection of numerical chromosomal aberrations in paraffin-embedded malignant pleural mesothelioma by non-isotopic *in situ* hybridization. *J Pathol* **175**: 219–226

Taguchi T, Jhanwar SC, Siegfried JM, Keller SM and Testa JR (1993) Recurrent deletions of specific chromosomal sites in 1p, 3p, 6q, and 9p in human malignant mesothelioma. *Cancer Res* **53**: 4349–4355

Tiainen M, Tammilehto L, Rautonen J, Tuomi T, Mattson K and Knuutila S (1989) Chromosomal abnormalities and their correlations with asbestos exposure and survival in patients with mesothelioma. *Br J Cancer* **60**: 618–626

Tiainen M, Hopman A, Moesker O, Ramaekers F, Wessman M, Laasonen A, Pyrhönen S, Tammilehto L, Mattson K and Knuutila S (1992) Interphase cytogenetics on paraffin sections of malignant pleural mesothelioma. A comparison to conventional karyotyping and flow cytometric studies. *Cancer Genet Cytogenet* **62**: 171–179

Van der Meeren A, Seddon MB, Kispert J, Harris CC and Gerwin BI (1993*a*) Lack of expression of the retinoblastoma gene is not frequently involved in the genesis of human mesothelioma. *Eur Respir Rev* **3**: 177–179

Van der Meeren A, Seddon MB, Betsholtz CA, Lechner JF and Gerwin BI (1993*b*) Platelet-derived growth factor A-chain overexpression is associated with tumorigenic conversion of human mesothelial cells. *Eur Respir Rev* **3**: 180–185

Wagner JC, Sleggs CA and Marchand P (1960) Diffuse pleural mesotheliomas and asbestos exposure in the Northwestern Cape Province. *Br J Ind Med* **17**: 260–271

Xiao S, Li D, Vijg J, Sugarbaker DJ, Corson JM and Fletcher JA (1995) Codeletion of *p15* and *p16* in primary malignant mesothelioma. *Oncogene* **11**: 511–515

Zeiger MA, Gnarra JR, Zbar B, Linehan WM and Pass HI (1994) Loss of heterozygosity on the short arm of chromosome 3 in mesothelioma cell lines and solid tumors. *Genes Chrom Cancer* **11**: 15–20

© Cancer Research Campaign 1997